# Manual long axis strain compared to automated feature-tracking deformation imaging for identification of masked left heart involvement in pulmonary hypertension

**DOI:** 10.1007/s10554-026-03664-2

**Published:** 2026-02-26

**Authors:** Ben N. Schmermund, Andreas J. Rieth, Matthias Rademann, Pauline C. Wilke, Steffen D. Kriechbaum, Jan S. Wolter, Andreas Schuster, Christoph B. Wiedenroth, Alexander Schulz, Julia M. Treiber, Samuel Sossalla, Andreas Rolf, Sören J. Backhaus

**Affiliations:** 1 Department of Cardiology, Campus Kerckhoff of the Justus-Liebig-University Giessen, Kerckhoff-Klinik, Bad Nauheim, Germany; 2 Cardio-Pulmonary Institute (CPI), Giessen/Bad Nauheim, Germany; 3 FORUM Medicine, Cardiology, Rosdorf, Germany; 4 Department of Cardiology and Pneumology, University Medical Center Göttingen, Georg- August University, Göttingen, Germany; 5 German Center for Cardiovascular Research (DZHK), Partner Site Lower Saxony, Göttingen, Germany; 6 Department of Thoracic Surgery, Campus Kerckhoff of the Justus-Liebig-University Giessen, Kerckhoff-Clinic, Bad Nauheim, Germany; 7 Department of Medicine, Cardiovascular Division, Beth Israel Deaconess Medical Center, Harvard Medical School, Boston, MA USA; 8 Department of Cardiology and Angiology, Medical Clinic I, University Hospital Giessen, Justus-Liebig-University Giessen, Giessen, Germany

**Keywords:** Pulmonary hypertension, Left heart involvement, Feature-tracking, Strain, Manual long axis strain

## Abstract

**Background:**

Left heart disease (LHD) may cause or coexist in pulmonary hypertension (PH). Left atrial (LA) deformation identifies LHD in PH, however, feature-tracking (FT) deformation imaging remains underused due to limited inter-vendor agreement. Manual long axis strain (LAS) has been introduced as a software independent approach for deformation assessment. Consequently, we sought to compare their diagnostic accuracies for identification of LHD in PH.

**Methods:**

Patients referred to both right heart catheterisation (RHC) and cardiovascular magnetic resonance (CMR) imaging were enrolled in this monocentric registry. Patients were classified by RHC according to current guideline recommendations. CMR assessment included FT-deformation imaging for LA total strain (Es) and left ventricular (LV) global longitudinal strain (GLS) as well as LV/LA LAS.

**Results:**

In total *n* = 209 PH patients were included with *n* = 126 undergoing exercise-stress testing (*n* = 55 normal, *n* = 72 pre-capillary, *n* = 27 combined post-/precapillary, *n* = 15 isolated postcapillary, *n* = 34 exercise and *n* = 6 unclassified PH). LAS showed strong correlation with FT for both LA (*r* = 0.78, *p* < 0.001) and LV (*r* = 0.83, *p* < 0.001) deformation. LA (AUC 0.81 vs. 0.81, *p* = 0.89) and LV LAS (AUC 0.75 vs. 0.77, *p* = 0.55) equally identified LHD at rest compared to their respective FT counterpart. LA LAS showed good diagnostic performance for identification of LHD unmasked during exercise stress for pulmonary capillary wedge pressure (PCWP) ≥25mmHg (AUC 0.73 vs. 0.79, *p* = 0.042) and PCWP/Cardiac Output (CO) Slope > 2 (AUC = 0.66 vs. 0.73, *p* = 0.045) but significantly inferior to LA Es. LV LAS showed similar performance compared to LV GLS, but worse compared to LA LAS.

**Conclusion:**

Software-independent LAS shows strong correlation to its FT counterparts and good diagnostic accuracy for detection of LHD in PH. However, for detection of masked LHD detected during exercise-stress only, FT LA Es remains the most accurate parameter.

## Introduction

Pulmonary hypertension (PH) is on the rise and associated with high morbidity and mortality already at early disease stages^1^, ^2^. Beyond the underlying mechanistic categories comprised in the world health organization (WHO) classification, hemodynamically, PH is classified into isolated post-capillary (IpcPH), pre-capillary, combined post-/pre-capillary (CpcPH), exercise and unclassified PH. The most common cause for PH is left heart disease (LHD)^3^. While WHO group 2 is defined as PH caused by LHD, all other groups may also feature coexisting LHD. As early diagnosis of left heart involvement and correct classification of PH hemodynamics have an impact on therapeutic management and its effectiveness, identification of LHD is key 3.

Over the past decade, deformation imaging has demonstrated superior prognostic value compared to conventional assessment of ejection fraction (LVEF) in ischemic and non-ischemic heart disease including heart failure with preserved and reduced ejection fraction (HFpEF/HFrEF) 4–7. In addition, left atrial strain (LA Es) has emerged as a reliable parameter in heart failure and following myocardial infarction with incremental value compared to ventricular functional assessment 8–10. In PH, LA Es has been shown to identify LHD unmasked during exercise stress superior to GLS^11^. However, feature-tracking (FT) is software dependent with vendor-specific reference values and not widely available. This has slowed clinical implementation in the past 12, 13. Alternatively, manual long axis strain (LAS) has been proposed to mirror FT derived longitudinal deformation assessment. LV LAS demonstrated similar results to LV global longitudinal strain (GLS) for event prediction following myocardial infarction 14, 15. Therefore, we aimed to investigate LA and LV LAS across the PH spectrum and to compare the diagnostic accuracies of manual LAS to automated FT for detection of LHD in PH.

## Methods

### Patient population

Patients were prospectively recruited to the Kerckhoff-Klinik BioCVI register after referral for CMR imaging between January 2017 and November 2023. This sub-study is comprised of patients who have also been referred for right heart catheterization (RHC) as part of the clinical routine. Clinical indications for CMR comprised assessment of myocardial function, inflammation, ischemia, and viability testing as well as cardiomyopathy evaluation. Clinically significant intracardial or veno-arterial shunts as well as valvular heart disease were criteria for exclusion.

### Right heart catheterization

Right heart catheterization (RHC) was performed through right internal jugular access or the femoral vein under fluoroscopic guidance when jugular access was not possible. A standard protocol was used for all RHC comprised of systolic, diastolic and median pressures in position of the pulmonary artery (PA) including pulmonary capillary wedge (PCWP), as well as the right atrium (RA) and ventricle (RV). Peripheral blood pressure was assessed non-invasively. Cardiac output (CO) was evaluated by the means of thermodilution or Fick principle. In case of clinically indicated and tolerated stress testing, exclusively thermodilution was used during rest and exercise-stress for CO quantification. PH was classified according to the 2022 ESC Guidelines as mPAP > 20mmHg. Precapillary PH is defined as PVR > 2 WU and PCWP ≤ 15 mmHg, IpcPH as PVR ≤ 2 WU PCWP > 15mmHg and CpcPH as PVR > 2 WU and PCWP > 15 mmHg. Exercise PH is defined as mPAP/CO slope rest to exercise > 3 mmHg/L/min and unclassified PH in case of mismatch for PVR and PCWP. Invasively determined LHD was defined as PCWP ≥ 15 mmHg at rest and/or ≥ 25mmHg during stress according to ESC guidelines for invasive diagnosis of HFpEF, as well as PCWP/CO slope > 2 16, 17.

### Cardiac magnetic resonance imaging

CMR imaging was conducted in head-first supine position on a clinical 3.0 Tesla Skyra (Siemens Healthineers, Erlangen, Germany) using an 18-array coil. Commercially available software was used for post-processing analyses (CVI42, Calgary, Canada). The clinical protocol followed the Society of Cardiovascular Magnetic Resonance guidelines and included balanced steady-state free precession (bSSFP) cine sequences in 2/3- and 4-chamber views (Ch) long-axis (LAX) orientations, and a short-axis stack (SAX) covering both ventricles. Right and left ventricular (LV) volumetric analyses were conducted on the SAX stack, including end-diastolic/systolic and stroke volumes (EDV/ESV/SV), EF as well as LV mass 18.

Automated deformation imaging comprised LV global longitudinal strain (GLS) and LA strain classified according to reservoir (Es), passive conduit (Ee) and active booster pump (Ea) function. LV GLS was assessed on 2-, 3- and 4-Ch. LA strain was assessed using biplanar feature tracking on 4- and 2-Ch. Ee and Ea were not assessed when imaging was done under ongoing atrial arrhythmia^19^.

**Fig. 1:**
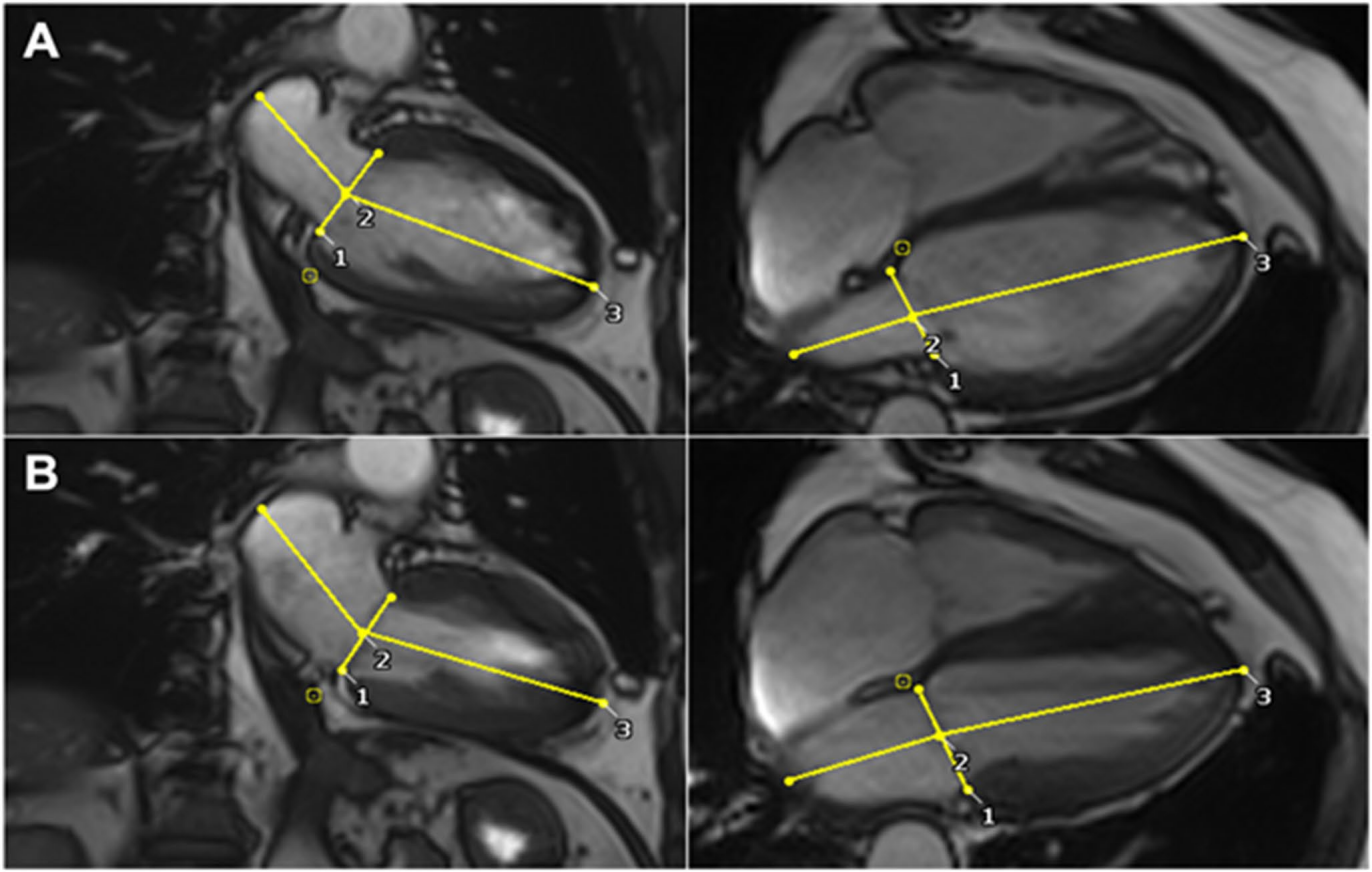
Fast manual long axis strain (LAS) for the left ventricle and atrium (LV/LA). The LA is measured from the mitral valve plane to the furthest atrial point excluding pulmonal arteries. The LV is measured from mitral valve plane to epicardial apex. A: Diastole and B: Systole

Manual LAS was assessed on 2-Ch and 4-Ch. For quantification of LA LAS, the distance from the middle of the mitral valve plane to the furthest atrial wall, excluding pulmonary veins, was measured in systole and diastole^20^. Similarly, for LV LAS, the distance from the middle of the mitral valve plane to the epicardial apical border was measured in systole and diastole^14^. Subsequently, the shortening was calculated in per cent **(**Fig. 1**)**.

### Statistical analyses

Parameters were tested for normal distribution using the Kolmogorow-Smirnow-test and are presented as median with interquartile range (IQR). Correlation was tested using Spearman’s correlation. Pairwise comparisons were done using Mann-Whitney U Test and comparison of more than two groups was done using Kruskal-Wallis Test with Bonferroni corrections. To compare diagnostic performance receiver operating curves (ROC) were calculated and area under the curves (AUC) compared using the non-parametric approach modelled after DeLong^21^. Calculations were performed on SPSS (Version 29.0.2.0 IBM, Armonk, New York, USA) and MedCalc (Version 23.3.7, MedCalc Software Ltd, Ostend, Belgium). Significant p-value was defined as < 0.05.

## Results

### Study population

**Fig. 2:**
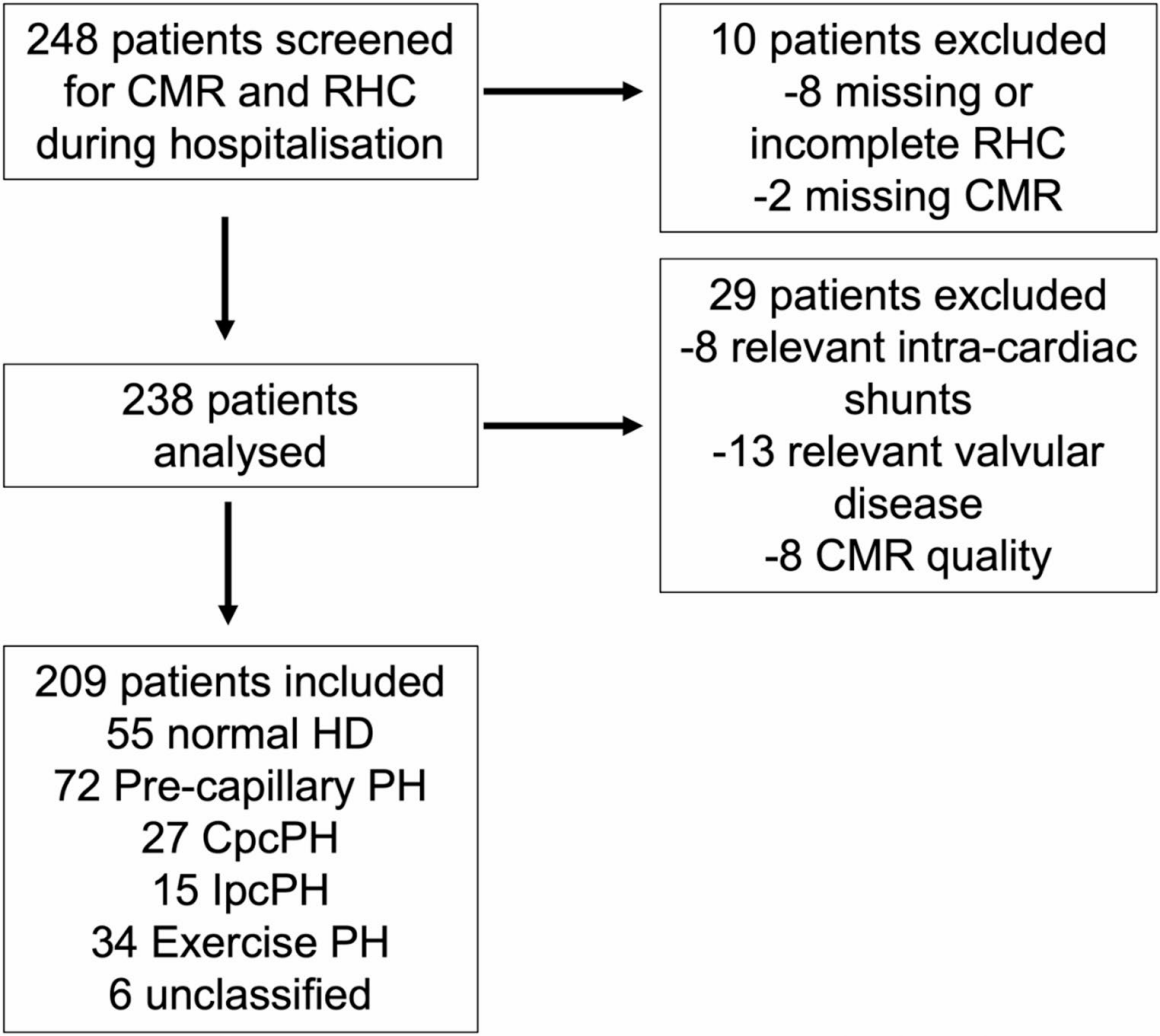
Flow diagram detailing patients screened, excluded and included. CMR: cardiac magnetic resonance, RHC: right-heart catheterization, HD: hemodynamics, PH: pulmonary hypertension, CpcPH: combined post-precapillary PH, IpcPH: isolated postcapillary PH

Baseline characteristics and flow diagram detailing patient screening are given in Table 1; Fig. 2. The final population consisted of *n* = 209 patients (*n* = 55 normal, *n* = 72 pre-capillary, *n* = 27 CpcPH, *n* = 15 IpcPH, *n* = 34 exercise and *n* = 6 unclassified PH). *N* = 126 patients underwent exercise-stress RHC, during which *n* = 38 showed PCWP ≥ 25mmHg (17 of which had a PCWP < 15mmHg at rest) and 69 showed PCWP/CO > 2. Median time between RHC and CMR was 1 day (IQR 1–3 days). Patients’ median age was 62 years and 43% were female. RHC results are shown in Tables 2 and 3.

**Table 1:** Patient population, median and interquartile range [IQR] shown. TAPSE (*n* = 178) and NT pro-BNP (*n* = 176) were not available for every patient. IPC/Pc/CPC PH: isolated post capillary/pre-capillary/combined post- and pre-capillary pulmonary hypertension, BMI: body mass index, LVEF: left ventricular ejection fraction, GFR: glomerular filtration rate, TAPSE: tricuspid annular plane systolic excursion. Parts of this table have been published previously Schmermund et al. 2025^11^

	Total	Normal	IpcPH	PcPH	CpcPH	Exercise	Unclassified
n =	209	55 (26%)	15 (7%)	72 (34%)	27 (13%)	34 (16%)	6 (3%)
n exercise RHC	126 (62%)	20 (36%)	8 (53%)	47 (65%)	13 (48%)	34 (100%)	4 (66%)
Female	89/209 (43%)	25 (45%)	7 (46%)	29 (40%)	9 (33%)	17 (50%)	2 (33%)
Age (years)	62 [50–71]	53 [39–64]	63 [54–74]	65 [52–72]	67 [38–74]	65 [54–74]	64 [60–79]
BMI (kg/m²)	27.4 [23.6–30.9]	27.7 [23.8–31.6]	26.9 [22.6–29.7]	26.7 [22.1–30.4]	26.5 [23.4–30.5]	27.7 [23.9–30.3]	39.0 [29.0–45.3.0.3]
LVEF ≥ 50%	162 (78%)	50 (91%)	8 (53%)	59 (82%)	13 (48%)	26 (76%)	6 (100%)
LVEF 40–50%	23 (11%)	1 (2%)	3 (20%)	9 (1%)	5 (19%)	5 (15%)	0
LVEF < 40%	24 (12%)	4 (7%)	4 (26%)	4 (6%)	9 (33%)	3 (9%)	0
Atrial Arrhythmia	63 (30%)	12 (22%)	11 (73%)	10 (14%)	15 (56%)	12 (35%)	3 (50%)
Diabetes mellitus	37/209 (18%)	5 (9%)	3 (20%)	11 (15%)	10 (37%)	5 (15%)	3 (50%)
GFR (ml /min/1,73m²)	84.34 [62.50–103.83.50.83]	94.42 [73.91–111.91.91.91]	71.30 [53.94–102.34.94.34]	84.82 [62.17–104.30]	64.82 [62.62–98.84]	80.40 [63.83–90.91]	72.52 [62.62–98.84]
TAPSE (mm)	18 [15–21]	18 [17–20]	17 [13–21]	18 [15–21]	15 [12–22]	18 [16–22]	22 [19–25]
NT pro-BNP (pg/dl)	596 [140–2015]	171 [56–566]	2352 [1143–6232]	613 [236–1337]	2286 [1783–7011]	287 [103–1012]	1526 [448–1837]

**Table 2:** RHC values at rest (n=209) and exercise stress (n=126). Metrics shown as median and interquartile range [IQR]. IPC/Pc/CPC PH: isolated post capillary/pre-capillary/combined post- and pre-capillary pulmonary hypertension, LV: left ventricle. LA: left atrium, EF: ejection fraction, GLS: global longitudinal strain, LAS: long axis strain, es: reservoir. p- values showing differences between all groups. Parts of this table have been published previously Schmermund et al. 2025^11^

	Total	Normal	IpcPH	PcPH	CpcPH	Exercise	Unclassified	*p*-value
Rest mPAP (mmHg)	23.0 [18.0–31.0]	16.0 [14.0–18.0]	28.0 [25.0–30.0]	29.5 [IQR 19.8]	35.0 [31.0–41.0]	18.5 [15.8–19.3]	23.0 [21.8–24.3]	< 0.001
Exercise mPAP (mmHg)	43.0 [36.0–53.0]	30.5 [27.3–35.8]	42.5 [37.3–52.3]	52.0 [46.0–61.0]	54.0 [49.0–59.5.0.5]	37.0 [30.0–42.0]	39.5 [32.3–45.3]	< 0.001
Rest PCWP (mmHg)	10.0 [8.0–14.0]	8.0 [6.0–10.0]	22.0 [18.0–24.0]	11.0 [8.0–13.0]	21.0 [18.0–25.0]	9.0 [6.8–11.3]	11.5 [9.3–14.0]	< 0.001
Exercise PCWP (mmHg)	19.0 [15.0–25.0]	15.0 [11.0–17.0]	31.0 [27.8–35.5]	17.0 [13.0–22.0]	28.5 [20.0–33.8.0.8]	20.0 [16.5–25.5-0]	21.0 [17.0–25.8.0.8]	< 0.001
Rest CI (l/m^2^)	2.37 [1.91–2.78]	2.59 [2.00–2.93.00.93]	2.25 [1.87–2.54]	2.38 [2.08–2.89]	1.81 [1.42–2.37]	2.22 [2.00–2.60.00.60]	2.80 [2.35–3.32]	< 0.001
Exercise CI (l/m^2^)	3.91 [2.99–5.20]	5.89 [5.02–6.85]	2.41 [1.88–3.62]	3.78 [3.07–5.15]	2.61 [2.11–3.64]	3.76 [3.04–4.52]	4.81 [3.58–5.21]	< 0.001
Rest PVR (WU)	2.47 [1,72-3.84]	1.62 [1.07–2.21]	1.62 [1.36–1.92]	3.82 [2.84–6.49]	4.06 [2.79–5.00.79.00]	1.89 [1.54–2.48]	1.74 [1.62–1.95]	< 0.001
Exercise PVR (WU)	2.60 [1.83–4.20]	1.57 [0.97–1.83]	2.70 [1.60–3.43]	4.26 [2.87–6.67]	4.32 [3.29–8.89]	2.25 [1.57–2.73]	1.64 [1.46–1.92]	< 0.001
mPAP/CO (mmHg/l)	7.20 [3.76–11.78]	2.56 [1.83–2.87]	11.27 [9.67–14.33]	9.02 [5.75–14.19]	10.95 [2.95–22.57]	6.60 [5.16–9.39]	3.68 [2.71–7.29]	< 0.001
PCWP/CO (mmHg/l)	2.58 [0.76–6.11]	0.86 [0.57–1.54]	7.16 [4.29–8.84]	2.31 [0.77–6.29]	10.95 [(-)17.83–6.78]	3.62 [2.43–6.44]	3.21 [1.21–5.57]	< 0.001

### Software dependent FT deformation imaging vs. manual LAS

**Fig. 3:**
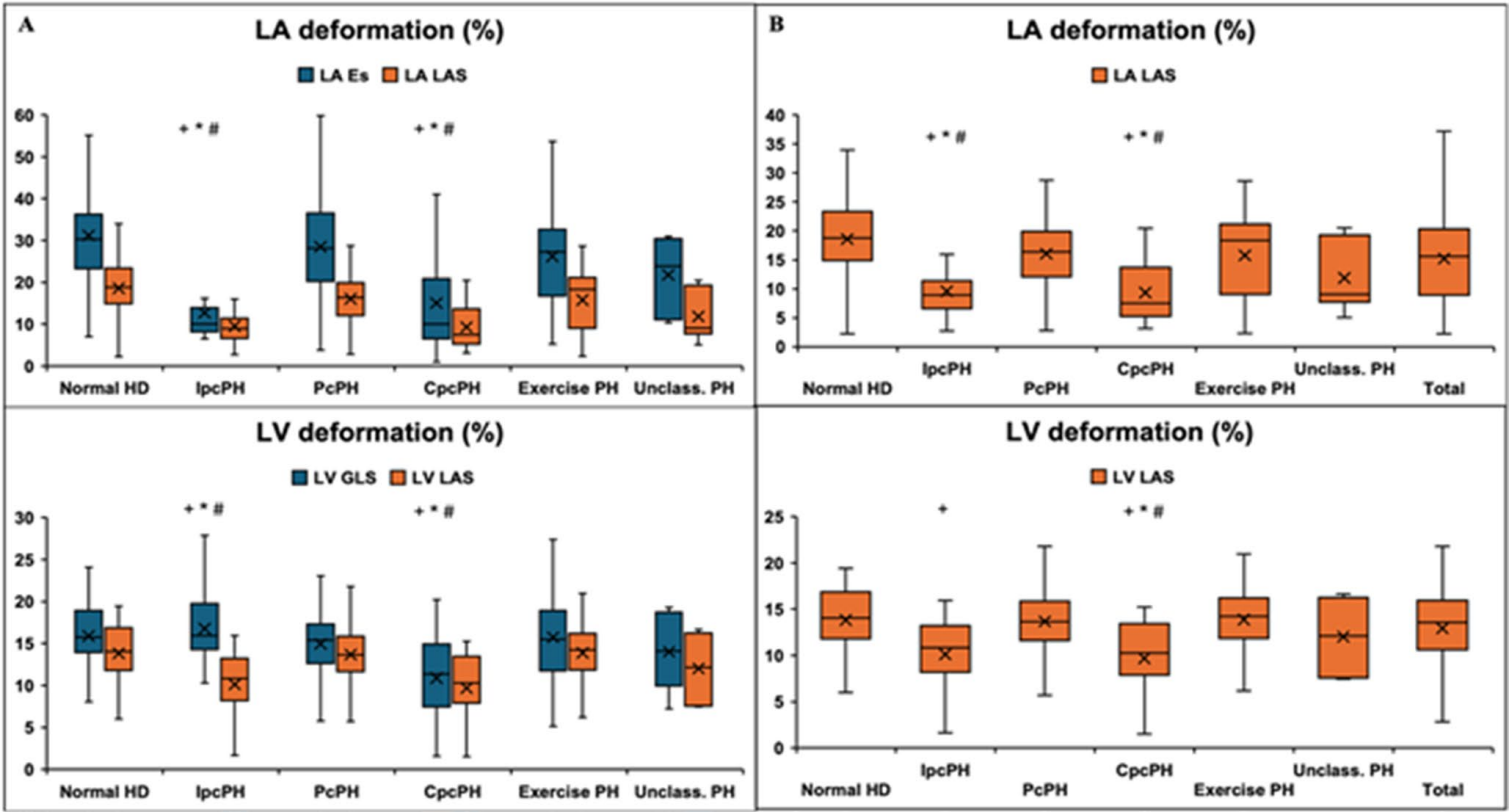
Boxplots showing LA and LV deformation data across the PH spectrum. Feature-tracking derived LA Es and manual LA LAS show similar differences between PH groups, whereas LV GLS and LV LAS do not. **A** shows LA Es and LV GLS in blue and surrogate parameters LA and LV LAS in comparison in orange. **B** shows LA and LV LAS in orange. Significant differences *p* < 0.05 are indicated compared to normal hemodynamics by +, to PcPH by * and exercise PH by #. IPC/Pc/CPC PH: isolated post capillary/pre-capillary/combined post- and pre-capillary pulmonary hypertension, LA Es: Left atrial reservoir strain, LV EF/GLS: Left ventricular ejection fraction/global longitudinal strain, LAS: Long axis strain. LV values shown as absolutes for better presentation

**Fig. 4:**
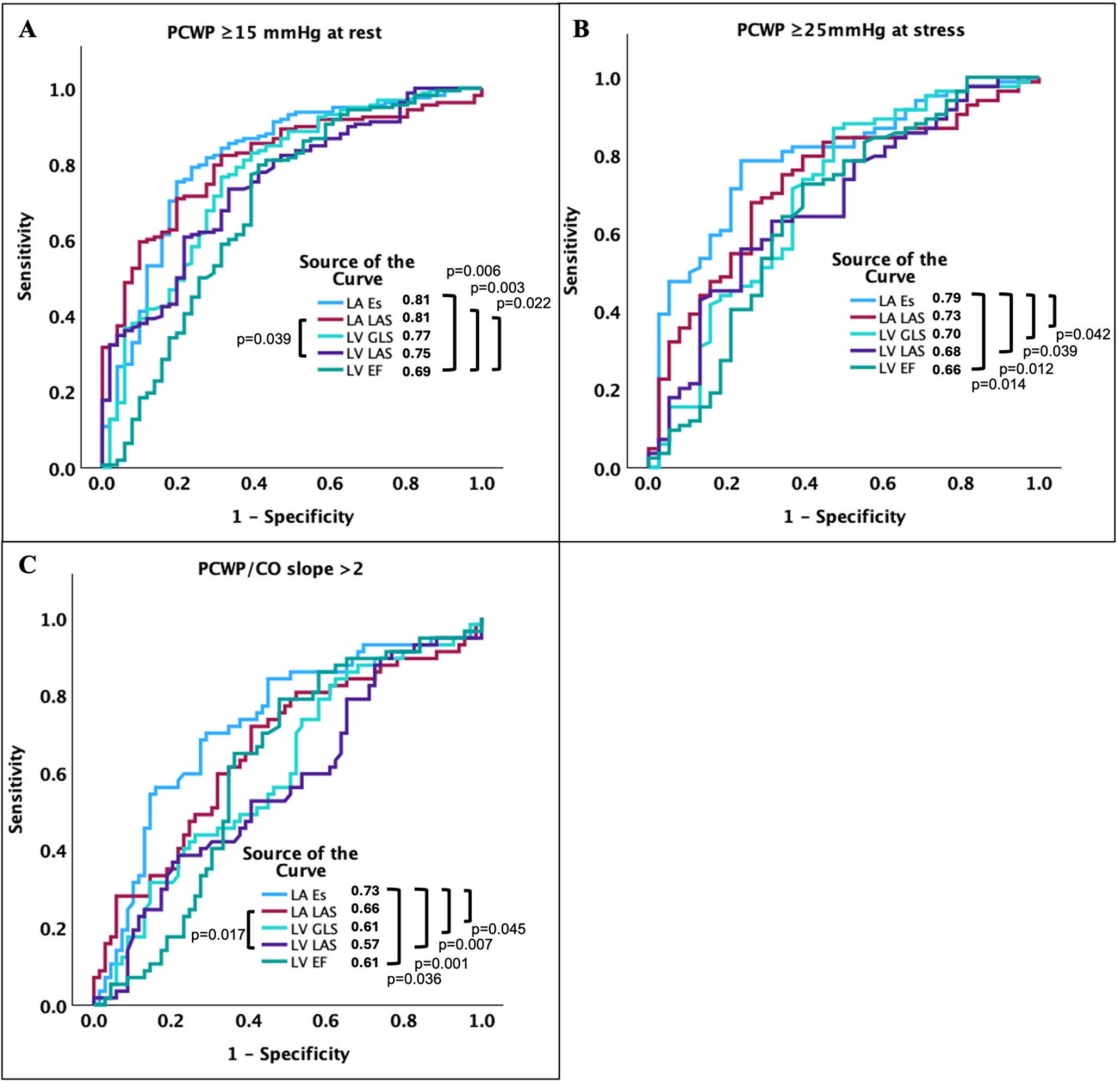
Diagnostic performance of functional parameters to identify invasively diagnosed left heart disease. **A**: LA Es and LA LAS show similar performance for identification of pulmonary capillary wedge pressure (PCWP) ≥15mmHg at rest **B**: LA Es shows superior performance for identification of stress PCWP ≥ 25mmHg **C**: LA Es shows superior performance in identifying PCWP/cardiac output (CO) slope ≥ 2.LA Es: left atrial reservoir strain, LV EF/GLS: left ventricular ejection fraction/global longitudinal strain, LAS: long axis strain

Results for deformation imaging across the PH spectrum are shown in Fig. 3 and for diagnostic performance in Fig. 4 for LA reservoir strain (LA Es), LA manual long axis strain (LA LAS), LV global longitudinal strain (LV GLS) and LV manual long axis strain (LV LAS).

### Left ventricular analyses

LV LAS was lowest in CpcPH and showed significant differences to normal hemodynamics.

(−10.3% vs. −14.0%, *p* = 0.001), precapillary PH (−13.7%, *p* = 0.003) and exercise PH (−14.2%, *p* = 0.003). IpcPH showed lower LV LAS compared to normal hemodynamics (−10.8% vs.

−14.0%, *p* = 0.029). LV LAS and GLS showed a strong correlation (0.81, *p* < 0.001). LV LAS and GLS had a similar AUC for identification of patients with PCWP ≥15mmHg at rest (0.75 vs. 0.77, *p* = 0.55), ≥25mmHg under stress testing (0.68 vs. 0.70, *p* = 0.59), and PCWP/CO > 2 (0.57 vs. 0.61, *p* = 0.25).

### Left atrial analyses

LA LAS was lowest in patients with CpcPH (7.5%) and IpcPH (8.9%). Both were significantly lower compared to normal hemodynamics (18.8%, both *p* < 0.001), pre-capillary PH (16.3%, *p* = 0.002/0.019) and exercise PH (18.4%, *p* < 0.001/0.028). LA LAS and Es showed a strong correlation (0.78, *p* < 0.001). LA LAS and Es had similar AUC for identification of patients with PCWP ≥15mmHg at rest (AUC 0.81 vs. 0.81, *p* = 0.89) which were significantly larger compared to LVEF (AUC 0.69, *p* = 0.003/0.006). LA LAS showed good but statistically inferior performance compared to Es in identification of patients with PCWP ≥25mmHg during exercise-stress (AUC 0.73 vs. 0.79, *p* = 0.042) or with a PCWP/CO slope > 2 (AUC 0.66 vs. 0.73, *p* = 0.045).

### Atrial phasic functional analyses

LA LAS showed higher diagnostic accuracy for LHD in PH at rest compared to LA Ee (AUC 0.82 vs. 0.74, *p* = 0.002) and similar compared to LA Ea (AUC 0.82, *p* = 0.77). Diagnostic accuracies for LA LAS and Ee/Ea were similar for identification of LHD in PH unmasked during exercise stress. AUC and comparisons with associated p-values are reported in Tables 4 and 5, specificity and sensitivity for Youden indices in Table 6).

**Table 3:** Metrics shown as median and interquartile range [IQR]. IPC/Pc/CPC PH: isolated post capillary/pre-capillary/combined post- and pre-capillary pulmonary hypertension, LV: left ventricle. LA: left atrium, EF: ejection fraction, GLS: global longitudinal strain, LAS: long axis strain, Es: reservoir. p- Values showing differences between all groups. Parts of this table have been published previously Schmermund et. al. 2025^11^.

	Total	Normal	IpcPH	PcPH	CpcPH	Exercise	Unclassified	*p*-value
LVEF (%)	57.0 [50.8–62.8]	57.1 [54.6–62.8]	52.9 [36.7–58.3]	57.3 [53.0–63.4.0.4]	50.8 [35.2–64.2]	57.9 [50.0–63.9.0.9]	61.5 [58.8–65.9]	0.05
LV GLS (%)	−15.2 [−18.0−12.0]	−15.7 [−18.9−13.9]	−11.3 [−14.4−8.0]	−15.7 [−18.1−14.1]	−11.4 [−15.5−7.5]	−15.5 [−18.9−11.8]	−14.1 [−18.7−10.0]	< 0.001
LV LAS (%)	−13.6 [−15.9−10.7]	−14.0 [−16.9−11.8]	−10.8 [−13.2−8.2]	−13.7 [−15.9−11.6]	−10.3 [−13.4−7.9]	−14.2 [−16.2−11.8]	−12.1 [−16.2−7.6]	< 0.001
LA Es (%)	26.7 [13.8–34.7]	30.8 [23.4–37.3]	10.0 [8.2–13.9]	28.2 [20.3–36.5]	10.0 [6.6–20.8]	26.9 [16.8–32.0]	23.9 [11.1–30.4]	< 0.001
LA LAS (%)	15.6 [8.9–20.4]	18.8 [14.9–23.4]	8.9 [6.6–11.4]	16.3 [12.1–19.9]	10.3 [7.9–13.44.9.44]	18.4 [9.0–21.2.0.2]	9.0 [7.7–19.3]	< 0.001

**Table 4:** Pairwise comparison and AUCs for diagnostic accuracy. PCWP: pulmonary capillary wedge pressure, CO: cardiac output, LA Es/LAS: left atrial reservoir/long axis strain, LV EF/GLS/LAS: left ventricular ejection fraction/global longitudinal strain/long axis strain

	AUC	AUC Difference	*p*-value	95% confidence interval
Rest PCWP ≥ 15mmHg				
LA LAS - LA Es	0.81 vs. 0.81	0.00	0.89	(-)0.05–0.06
LA LAS - LVEF	0.81 vs. 0.69	0.12	0.003	(-)0.20 - (-)0.04
LA LAS - LV GLS	0.81 vs. 0.77	0.05	0.20	(-)0.02–0.11
LA LAS - LV LAS	0.81 vs. 0.75	0.06	0.039	0.26 − 0.003
LA Es - LVEF	0.81 vs. 0.69	0.12	0.006	(-)0.22 - (-)0.04
LA Es - LV GLS	0.81 vs. 0.77	0.04	0.20	(-)0.03–0.12
LA Es - LV LAS	0.81 vs. 0.75	0.07	0.085	(-)0–01–0.14
LV GLS - LVEF	0.77 vs. 0.69	0.08	0.022	(-)0.14 - (-)0.01
LV GLS - LV LAS	0.77 vs. 0.75	0.02	0.55	(-)0.08 − 0.04
LV LAS - LVEF	0.75 vs. 0.69	0.06	0.06	(-)0.13 − 0.02
Stress PCWP ≥ 25mmHg				
LA LAS - LA Es	0.73 vs. 0.79	0.04	0.042	0.002–0.12
LA LAS - LVEF	0.73 vs. 0.66	0.07	0.20	(-)0.18 − 0.04
LA LAS - LV GLS	0.73 vs. 0.70	0.03	0.51	(-)0.07–0.14
LA LAS - LV LAS	0.73 vs. 0.68	0.05	0.24	(-)0.04–0.14
LA Es - LVEF	0.79 vs. 0.66	0.13	0.014	(-)0.24 - (-)0.03
LA Es - LV GLS	0.79 vs. 0.70	0.09	0.039	0.01–0.19
LA Es - LV LAS	0.79 vs. 0.68	0.11	0.012	0.03–0.21
LV GLS - LVEF	0.70 vs. 0.66	0.04	0.47	(-)0.14 − 0.06
LV GLS - LV LAS	0.70 vs. 0.68	0.02	0.59	(-)0.9 − 0.05
LV LAS - LVEF	0.68 vs. 0.66	0.02	0.75	(-)0.13 − 0.09
PCWP/CO slope ≥ 2				
LA LAS - LA Es	0.66 vs. 0.73	0.07	0.045	0.001–0.13
LA LAS - LVEF	0.66 vs. 0.61	0.05	0.38	(-)0.16 − 0.06
LA LAS - LV GLS	0.66 vs. 0.61	0.05	0.21	(-)0.14–0.32
LA LAS - LV LAS	0.66 vs. 0.57	0.09	0.017	0.02–0.17
LA Es - LVEF	0.73 vs. 0.61	0.12	0.036	(-)0.22 – (-) 0.01
LA Es - LV GLS	0.73 vs. 0.61	0.12	0.007	0.03–0.21
LA Es - LV LAS	0.73 vs. 0.57	0.16	0.001	0.07–0.25
LV GLS - LVEF	0.61 vs. 0.61	0.00	0.91	(-)0.09–0.11
LV GLS - LV LAS	0.61 vs. 0.57	0.04	0.25	(-)0.03–0.10
LV LAS - LVEF	0.57 vs. 0.61	0.04	0.43	(-)0.06–0.15

### Comparison of manual LV/LA LAS

Compared to LV LAS, LA LAS showed superior diagnostic performance for identification of LHD evident at rest (AUC 0.81 vs. 0.75, *p* = 0.039). In masked LHD, LA LAS showed numerically higher diagnostic accuracy compared to LV LAS for identification of PCWP ≥ 25mmHg during exercise stress (AUC 0.73 vs. 0.68, *p* = 0.24) reaching statistical significance for identification of patients with a PCWP/CO slope > 2 (AUC 0.66 vs. 0.57, *p* = 0.017).

## Discussion

The present manuscript expands the evidence on longitudinal deformation assessment comparing manual LAS to automated FT GLS with emphasis on LHD unmasked during exercise-stress. Firstly, manual LAS for LA and LV longitudinal deformation shows strong correlation to its respective counterpart FT Es or GLS. Secondly, it allows for similar diagnostic accuracy to detect LHD in PH evident at rest. Lastly, whilst LV LAS and GLS equally identify LHD unmasked during exercise stress, FT Es shows the highest diagnostic accuracy to detect masked LHD and emerges superior to manual LA LAS.

Considering the mismatch between the proven diagnostic and prognostic superiority of deformation imaging but slow clinical routine implementation, efforts have been directed towards fast, reliable and perhaps most importantly software independent metrics. LV LAS has shown predictive value for outcome following myocardial infarction and reliably discriminates healthy control patients compared to DCM, hypertrophic cardiomyopathy (HCM) and AL-Amyloidosis with higher accuracy compared to mitral annular plane systolic excursion (MAPSE) and LVEF 14, 15. LA LAS has shown prognostic value in HCM and in predicting outcome for patients with DCM^9^, ^10^. Our results reinforce the available literature on the ability of both LA and LV LAS to represent a surrogate parameter for FT-derived longitudinal deformation, given their strong correlation. Importantly, manual LAS achieves similar diagnostic accuracy to identify overt LHD (diagnosed at rest) in PH compared to their respective feature-tracking deformation imaging counterparts LA Es and LV GLS. Moreover, PH patients with left heart involvement according to RHC showed similar results for impairment of LA LAS and LA Es.

**Table 5:** Pairwise comparison and AUCs for diagnostic accuracy. Patients with atrial arrhythmia under imaging were excluded (n = 205). PCWP: pulmonary capillary wedge pressure, CO: cardiac output, LA Es/Ea/Ee/LAS: left atrial reservoir/booster pump/conduit strain/long axis strain

	AUC	AUC Difference	*p*-value	95% confidence interval
Rest PCWP ≥ 15mmHg				
LA Es - LA Ea	0.82 vs. 0.82	0.002	0.9	(-)0.03 − 0.013
LA Es - LA Ee	0.82 vs. 0.74	0.08	0.002	0.03–0.14
LA Es - LA LAS	0.82 vs. 0.81	0.01	0.78	(-)0.45 − 0.06
LA Ea - LA Ee	0.82 vs. 0.74	0.09	0.031	0.01–0.16
LA Ea - LA LAS	0.82 vs. 0.81	0.01	0.77	(-)0.53–0–72
LA Ee - LA LAS	0.74 vs. 0.81	0.08	0.012	(-)0.14 - (-)0.02
Stress PCWP ≥ 25mmHg				
LA Es - LA Ea	0.79 vs. 0.76	0.04	0.12	(-)0.01–0.08
LA Es - LA Ee	0.79 vs. 0.72	0.07	0.022	0.01–0.14
LA Es - LA LAS	0.79 vs. 0.73	0.06	0.042	0.002–0.12
LA Ea - LA Ee	0.76 vs. 0.72	0.04	0.46	(-)0.06–0.14
LA Ea - LA LAS	0.76 vs. 0.73	0.03	0.47	(-)0.05–0.10
LA Ee - LA LAS	0.72 vs. 0.73	0.01	0.79	(-)0.10 − 0.08
PCWP/CO slope ≥ 2				
LA Es - LA Ea	0.73 vs. 0.63	0.1	0.001	0.04 vs. 0.16
LA Es - LA Ee	0.73 vs. 0.73	0.003	0.93	(-)0.7 − 0.06
LA Es - LA LAS	0.73 vs. 0.66	0.07	0.045	0.001–0.13
LA Ea - LA Ee	0.63 vs. 0.73	0.1	0.07	(-)0.21 vs. 0.01
LA Ea - LA LAS	0.63 vs. 0.66	0.03	0.43	(-)0.11 vs. 0.05
LA Ee - LA LAS	0.73 vs. 0.66	0.07	0.11	(-)0.02 vs. 0.15

LA but not LV LAS emerged superior for identification of overt LHD identified at rest compared to LVEF. This is in line with LA LAS showing superiority compared to LV LAS. This finding stresses the value of atrial over ventricular longitudinal deformation. Moreover, left heart involvement can oftentimes only be uncovered by exercise-stress RHC (masked LHD)^22^, ^23^. Whilst LV LAS and GLS equally identified LHD unmasked during exercise-stress, especially exercise-stress LAS has demonstrated incremental value for early detection of HFpEF in the past 23. However, LA LAS remained inferior compared to FT derived LA Es opposed to LV longitudinal deformations assessments and its FT counterpart. This may arise from more complex atrial geometry, skewed axis for the LA in LV-centered image acquisition and subsequently more challenging overall deformation quantification^24^, ^25^. Statistical significance between LA LAS and LA Es for diagnostic accuracy of exercise induced LHD may be marginal, however this was consistent across both PCWP and PCWP/CO slope underlining robustness. An increment of 0.06 and 0.07 for PCWP and PCWP/slope respectively may also indicate clinically significant differences in diagnostic accuracy. FT imaging seems to provide more comprehensive and accurate information on atrial function covering the entire atrial wall, reducing the bias introduced by geometric complexity and LV centered images.

CMR FT is based on tracking of anatomical landmarks across the cardiac cycle. This does not require additional sequences opposed to CMR tagging and allows for more reliable quantification especially of the diastolic phase 12, 26, 27. Differentiation of LA strain into atrial phasic functions described by reservoir (Es), passive conduit (Ee) and active booster pump function (Ea) is possible. This phasic breakdown may provide more in-depth information about hemodynamic changes. Previously, von Roeder et al. demonstrated that LA Ee best predicts exercise intolerance in HFpEF 28. However, in our study population, LA Es shows overall best performance for identification of LHD in PH, with phasic functions of Ee and Ea showing statistically similar or worse value compared to LA LAS. Thus, distinction of atrial phasic function did not offer incremental diagnostic value.

**Table 6:** Sensitivity and specificity at maximum Youden Index for identification of invasively proven left heart involvement. Left atrial reservoir strain (LA Es) and manual long axis strain (LA LAS), as well as left ventricular global longitudinal strain (LV GLS) and LV LAS

Functional parameter	Youden Index	Sensitivity	Specificity
Rest PCWP ≥ 15mmHg			
LA Es	19.3%	0.791	0.765
LA LAS	14.0%	0.804	0.719
LV GLS	(-)14.0%	0.686	0.776
LV LAS	(-)12.0%	0.667	0.744
Exercise PCWP ≥ 25mmHg			
LA Es	23.1%	0.786	0.773
LA LAS	16.1%	0.737	0.679
LV GLS	(-)13.0%	0.526	0.869
LV LAS	(-)13.7%	0.684	0.631

LA Es has superior diagnostic accuracy for the identification of otherwise masked LHD during exercise-stress and may provide incremental value to the non-invasive diagnostic path for PH. At rest, LA LAS also shows good diagnostic performance equal to LA Es for detection of overt LHD. Opposed to earlier studies measuring semi-automated LA LAS, we focused on a software independent approach considering systole and diastole only. This software independent metric is easily applicable and has already demonstrated prognostic impact in heart failure across the EF spectrum following myocardial infarction. LA LAS offers fast, vendor- and software independent approach for approximation of LA longitudinal deformation. Besides LA Es, LA LAS offers second best detection of masked LHD at rest detected during exercise-stress RHC. Consequently, LA LAS emerges as an ideal screening metric to identify LHD at rest with a fair balance of diagnostic accuracy and clinical routine feasibility. It is readily available, easy to implement to the clinical routine and perhaps above all allows the validation of one specific reference range as opposed to vendor dependency in FT. Consequently, LA LAS may thus be used for decision making on subsequent referral for further diagnostics, such as RHC including exercise-stress testing 20.

### Limitations

This single-center study with a relatively small number of 209 patients should be considered hypothesis generating with findings needing additional external validation. Feature-tracking analyses were performed using one specific vendor. Vendor variability may apply. Moreover, no follow-up data was available resulting in the inability of comparing prognostic impact of manual LAS compared to dedicated deformation imaging in PH. Incomplete echocardiographic data sets did not allow for comparison of LAS between methods.

## Conclusion

Atrial function quantification emerged superior compared to ventricular function for identification of masked LHD identified during exercise-stress. With dedicated deformation imaging showing superior diagnostic value compared to manual LAS, FT underlines its clinical usefulness compared to the simple software independent approach of manual LAS.

## Data Availability

Regarding data availability, we confirm that all relevant data are within the paper and all data underlying the findings are fully available without restriction and can be accessed at the Kerckhoff Heart Research Institute (KHFI) by researchers who meet the criteria for access to confidential data.

## Abbreviations

AUC: Area Under The Curve
CI: Cardiac Index
CpcPH: Combined Post-/Pre-capillary Pulmonary Hypertension
CO: Cardiac Output
Ea: Booster Pump Strain
Ee: Conduit Strain
Es: Reservoir Strain
FT: Feature Tracking
GLS: Global Longitudinal Strain
HFpEF: Heart Failure With Persistent Ejection Fraction
HFrEF: Heart Failure With Reduced Ejection Fraction
IpcPH: Isolated Post-capillary Pulmonary Hypertension
LA: Left Atrium
LAS: Long Axis Strain
LHD: Left Heart Disease
LV: Left Ventricle
mPAP: Mean Pulmonary Artery Pressure
PcPH: Pre-capillary Pulmonary Hypertension
PCWP: Pulmonary Capillary Wedge Pressure
PH: Pulmonary Hypertension
PVR: Pulmonary Vascular Resistance
RA: Right Atrium
RHC: Right Heart Catherization
RV: Right Ventricle
TAPSE: Tricuspid Annular Plane Systolic Excursion
WU: Wood Units
